# Phthalate-Induced Liver Protection against Deleterious Effects of the Th1 Response: A Potentially Serious Health Hazard

**DOI:** 10.1155/2007/49671

**Published:** 2008-01-01

**Authors:** Mostafa Z. Badr, Alexander Shnyra, Mikhail Zoubine, Maxim Norkin, Betty Herndon, Tim Quinn, Roberto N. Miranda, Michael L. Cunningham, Agostino Molteni

**Affiliations:** ^1^Division of Pharmacology and Toxicology, School of Pharmacy, University of Missouri-Kansas City, Kansas City, MO 64108, USA; ^2^Department of Pharmacology and Microbiology, Kansas City University of Medicine and Biosciences, Kansas City, MO 64106, USA; ^3^Department of Basic Medical Sciences, School of Medicine, University of Missouri-Kansas City, Kansas City, MO 64108, USA; ^4^Department of Pathology, School of Medicine, University of Missouri-Kansas City, Kansas City, MO 64108, USA; ^5^Laboratory of Pharmacology and Chemistry, National Institute of Environmental Health Sciences, Research Triangle Park, NC 27709, USA

## Abstract

Infection with *Mycobacterium tuberculosis* (TB) induces pulmonary immunopathology mediated by classical Th1 type of acquired immunity with hepatic involvement in up to 80% of disseminated cases. Since PPAR agonists cause immune responses characterized by a decrease in the secretion of Th1 cytokines, we investigated the impact of activating these receptors on hepatic pathology associated with a well-characterized model of Th1-type pulmonary response. Male Fischer 344 rats were either maintained on a drug-free diet (groups I and II), or a diet containing diethylhexylphthalate (DEHP), a compound transformed in vivo to metabolites known to activate PPARs, for 21 days (groups III and IV). Subsequently, animals were primed with *Mycobacterium bovis* purified protein derivative (PPD) in a Complete Freund's Adjuvant. Fifteen days later, animals in groups II and IV were challenged with Sepharose 4B beads covalently coupled with PPD, while animals in groups I and III received blank Sepharose beads. Animals with Th1 response (group II) showed a marked structural disruption in the hepatic lobule. Remarkably, these alterations were conspicuously absent in animals which received DEHP (group IV), despite noticeable accumulation of T cells in the periportal triads. Immunostaining and confocal microscopy revealed hepatic accumulation of IFN$\gamma^{+}$ Th1 and IL-$4^{+}$ Th2 cells in animals from groups II and IV, respectively. Our data suggest a PPARα-mediated suppression of the development of a Th1 immune response in the liver, resulting in hepatoprotective effect. However, potentially negative consequences of PPAR activation, such as decreased ability of the immune system to fight infection and interference with the efficacy of vaccines designed to evoke Th1 immune responses, remain to be investigated.

## 1. INTRODUCTION

Although primary hepatic TB is an uncommon disease, pulmonary TB with hepatic involvement represents up to 80% of disseminated cases of this disease [1]. Reasons for the involvement of the liver in this disease are unclear. However, since pulmonary TB is known to evoke classical Th1-type of acquired immunity [2], it is conceivable that an overwhelming Th1-type immune reactions in the lungs may result in a systemic immune response which causes collateral damage to other organs, including the liver [3, 4].

PPARs are members of the steroid receptor superfamily [5]. Activation of the PPARα receptor subtype induces the secretion of the prototypic Th2 cytokine IL-4, while decreasing that of the prototypic Th1 cytokine, IFNγ, in both murine and human T cells [6]. Furthermore, in primary human hepatocytes and hepatoma cells, PPAR activators suppress IL-1-induced C-reactive protein as well as IL-6 induced fibrinogen expression, the major acute-phase response proteins in humans [7].

This study was therefore undertaken to (1) analyze whether experimentally-induced Th1-type immune response in the lungs, initiated by *Mycobacterium bovis*, may lead to immunopathological abnormalities in the liver, and (2) investigate the influence of activating PPAR receptors, which are abundant in the liver [8], on such hepatic response. We elected to use diethylhexylphthalate, one of the most abundant environmental pollutants which is transformed in the body to metabolites known to activate PPARs [8–15].

## 2. MATERIALS AND METHODS

### 2.1. Animals

Male Fischer 344 rats (250–300 gm body weight) were obtained from Taconic, Hudson NY. Upon arrival, animals were acclimatized for a week prior to experiment in an AAALC-accredited facility at the University of Missouri-Kansas City, on a daily cycle of alternating 12-hour periods of light and dark. All procedures were performed in compliance with relevant regulations.

### 2.2. Induction of Th1 response

Rats were randomly divided into 4 groups: groups I and II received a drug-free diet, while groups III and IV received a diet containing 12.000 ppm diethylhexylphthalate (DEHP) (Sigma Aldrich, St Louis, Missouri) for 21 days. Subsequently, rats in all groups received intraperitoneally 20 *μ*g of *Mycobacterium bovis* purified protein derivative (PPD) (Mycos Research, Loveland, Colorado) incorporated into 0.25 ml of complete Freund's adjuvant (CFA; Sigma Aldrich, St Louis, Missouri). Fifteen days later, animals in groups II and IV were challenged intravenously with approximately 6000 beads of Sepharose 4B covalently coupled with PPD, in 0.5 ml PBS. Animals in groups I and III received the Sepharose beads without PPD. All animals remained on their respective diets for additional 15 days, after which time they were sacrificed, and livers were removed and kept in neutralized 10% formalin until processing.

### 2.3. Histopathological and immunohistochemical studies

Tissue specimens were paraffin-embedded, and 4 *μ*m sections were deparaffinized and stained with hematoxylin and eosin. A semiquantitative evaluation of the histological damage to the liver was carried out according to previously published methodology [16]. Briefly, two pathologists, who were unaware of the treatments, objectively evaluated the tissue for the reported specific types of injury. A mean score was assigned to each slide ranging from 10, indicating the presence of definite damage, to 40, indicating very severe damage. A score of 5 indicated that some areas had specific damage whereas other areas were without morphological changes. Slides of tissues with normal appearance received a score of 0.

For immunohistochemical studies, slides were quenched using 3% hydrogen peroxide in methanol. Sections were then hydrated through gradient alcohols and phosphate-buffered saline. For antigen retrieval, slides were placed in neutralized target retrieval (Dako, Carpinteria, California) for 30 minutes in a steamer. Slides were then exposed for one hour, at room temperature, to primary antibodies: rabbit anti-CD3 (dilution 1 : 600, Dako), and mouse anti-CD79 (dilution 1 : 300, Neomarkers, Freemont, California). Blocking solution was used as a negative control on duplicate slides. Secondary antibody was biotinylated goat anti-immunoglobulins of mouse, rabbit, guinea pig, and rat primary antibodies. An avidin-biotin-peroxidase complex (Immunodetection System Biogenex, San Ramon, California) with DAB as a chromogen was used for detecting antibody binding.

### 2.4. Th1/Th2 hepatic responses

Liver tissue sections (8 *μ*m) were deparaffinized and processed for antigen retrieval by boiling in 10 mM citrate buffer (pH 6.0) for 20 minutes, cooled for 10 minutes, and then washed with PBS [16]. Subsequently, slides were conditioned with SuperBlock blocking buffer in PBS (Pierce, Rockford, Illinois) for 30 minutes at room temperature and were then incubated for 1 hour with the primary antibody (IL-4 and IFN-γ, Proscience, Inc., Poway, California) diluted in 10% SuperBlock blocking buffer according to the manufacturer's protocol. After incubation, slides were rinsed with PBS and washed with PBS (3×3 minute). The slides were then incubated for 1 hour with the secondary antibody (goat anti-mouse isotype-specific antibodies labeled with Alexa Fluor 488, or Alexa Fluor 568; Molecular Probes, Eugene, Oregon) diluted in 150 *μ*l of 10% SuperBlock blocking buffer. Slides were washed and mounted with Prolong Antifade Kit (Molecular Probes, Eugene, Oregon), protected with a cover slip, and stored in the dark until analysis by confocal microscopy. Background autofluorescence was controlled by using tissue sections which were processed as above, except the incubation with primary antibodies. Fluorescence was registered as 1.0 *μ*m optical sections in parallel in the 488 nm (green), or 568 nm (red) channels, using Nikon software EZ-C1, version 2.20, equipped with Ar/He/Ne lasers [17].

## 3. RESULTS

### 3.1. Effect of Th1 response on liver histopathology

**Figure 1:**
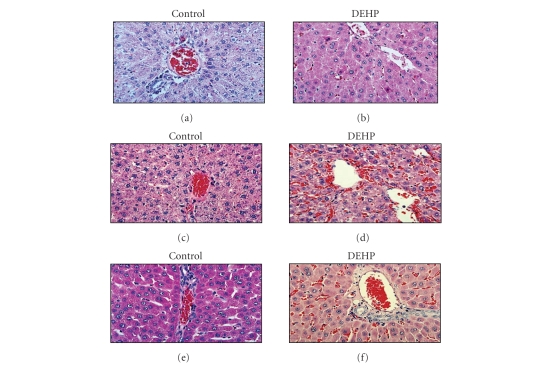
*Effect of DEHP on Th1-Induced damage of hepatocellular structure*. (a) Representative liver sections from rats fed either a drug-free diet; (b) DEHP-containing diet. (c) Animals on drug-free diet + naked sepharose beads, group I; (d) DEHP diet + naked sepharose beads, group III; (e) drug-free diet + PPD-coupled sepharose beads, group II; or (f) DEHP diet + PPD-coupled sepharose beads, group IV. Tissues from 5 animals per group were stained with hematoxylin and eosin following standard techniques. Photos are 100x.

Figure 1 shows representative hematoxylin and eosin-stained sections of livers from animals maintained on control, as well as DEHP-containing diets, in the absence of any further treatment. While livers of control animals did not show any morphological abnormality (see Figure 1(a)), dietary DEHP caused an intense eosinophilic cytoplasmic staining of hepatocytes, albeit without substantially altering the lobular structure of the organ (see Figure 1(b)). In addition, some cells demonstrated particularly intense eosinophilia and nuclei that were smaller and intensively hyperchromatic, with possible mitotic figures (see Figure 1(b)).

**Figure 2:**
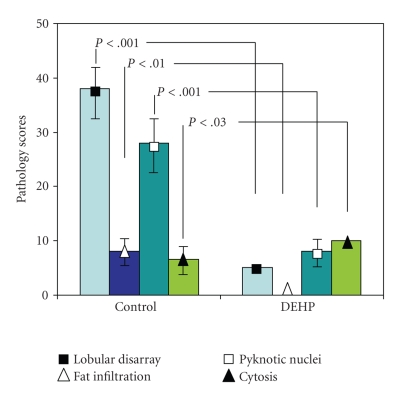
*Pathological evaluation of liver response to PPD*. Livers of rats fed either a drug-free diet + PPD-coupled sepharose beads, Control; or DEHP diet + PPD-coupled sepharose beads, DEHP were evaluated as described under Section 2. Results are means ± SD from 4-5 rats per group.

Immune response induced by PPD in animals maintained on control diet (groups I and II) resulted in the hepatic lobule disruption, vascular congestion, mild fatty infiltration, and appearance of many pyknotic nuclei (see Figures 1(c) and 1(e)). The damage was more severe in animals which received both soluble PPD and PPD-coupled beads (group II), with more evident parenchymal disruption, a larger number of pyknotic nuclei, and scattered inflammatory cells infiltrating the parenchyma (see Figure 1(e)). These alterations were absent in the livers of rats receiving the DEHP diet (see Figures 1(d) and 1(f)), where the lobular structure remained normal with modest infiltration of inflammatory cells in the periportal triads. A semiquantitative evaluation of the hepatic morphological alterations in all animal groups is summarized in Figure 2.

### 3.2. Hepatic immune response to *Mycobacterium bovis* PPD and shifting the Th1/Th2 balance in response to DEHP

**Figure 3:**
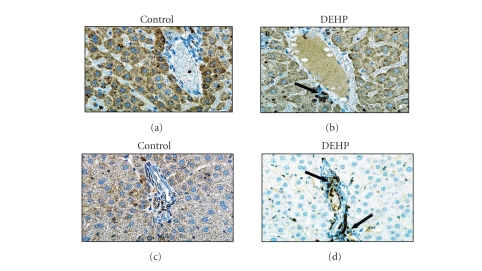
*Hepatic sublobular distribution of T cells in DEHP-treated rats.* (a) Representative liver sections from rats on drug-free diet + naked sepharose beads, group I; (b) DEHP diet + naked sepharose beads, group III; (c) drug-free diet + PPD-coupled sepharose beads, group II; or (d) DEHP diet + PPD-coupled sepharose beads, group IV. Tissues from 5 animals per group were stained with hematoxylin and eosin following standard techniques. Arrows point to representative T cells. Photos are 100x.

In livers of rats maintained on control diet, only scattered T (see Figure 3) and B (not shown) cells were observed following treatment with sepharose beads, in the presence or absence of *Mycobacterium bovis* PPD. The presence of T cells became evident in livers of rats maintained on the DEHP-containing diet, particularly in the portal triad of animals treated with PPD coupled beads (see Figure 3(d)).

**Figure 4:**
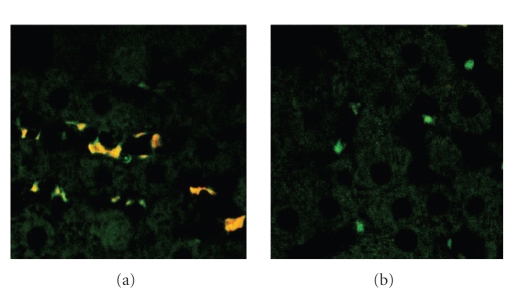
*Changes in liver-associated T cell cytokine pattern due to DEHP.* (a) Representative liver sections from rats fed either a drug-free diet + PPD-coupled sepharose beads; or (b) DEHP diet + PPD-coupled sepharose beads.

Double staining with antibodies against Th1-type cytokine IFN-γ (labeled with red fluorochrome) and Th2-type cytokine IL-4 (labeled with green fluorochrome) has revealed IL-4^+^/IFN-$\gamma^{+++}$ phenotype of liver-associated T cells in PPD-immunized animals kept on drug-free diet (see Figure 4(a)). In PPD-immunized animals receiving DEHP-diet, however, liver associated T cells were stained with bright green color indicating a full acquisition of IL-4^+++^/IFN-$\gamma^{-}$ (Th2) phenotype by the cells (see Figure 4(b)).

## 4. DISCUSSION

In human diseases and animal models, it has been demonstrated that specific immune responses mediated by the Th1-related cytokines are associated with resistance to infection caused by virsus and intracellular bacteria, while Th2-related cytokines exert opposing negative immunoregulatory functions [2]. However, since Th1 lymphocytes are major producers of IFNγ, a critical cytokine in the orchestration of potent pro-inflammatory response, it is conceivable that Th1 immune cells may not only contribute to the elimination of pathogens, but may also precipitate damage of host cells [3, 4].

### 4.1. PPARα, immune deviation, and hepatocellular protection

PPARs have been demonstrated to have regulatory effects on inflammatory and immune responses [5]. Indeed, agonists of these receptors have been shown to modulate cellular responses to a variety of inflammatory agents [5], as well as to alter immune cell functions [5, 6]. Activation of PPARα in cultures of murine or human T cells inhibited IFNγ, while promoting IL-4 secretion [6]. In mice, either of the PPARα agonists, gemfibrozil or fenofibrate inhibited clinical signs of Th1-mediated autoimmune encephalomyelitis [6].

In this study, evoking a Th1 response through exposure to PPD resulted in hepatic lobule disruption (see Figure 1). However, these morphological alterations were essentially absent in animals subjected to the same protocol, but receiving DEHP-containing diet. This hepatoprotective effect occurred concomitantly with a change in Th1/Th2 balance in liver-associated T lymphocytes. This effect can be characterized as a shift from the liver damaging IFNγ-controlled Th-1 response to the liver protective Th2 response associated with IL-4-positive lymphocytes (see Figure 4).

Simultaneous detection of IL-4 (green) and IFN-γ (red) by double fluorescence has revealed IL-4^+^/IFN-$\gamma^{+++}$ T cells identified by orange fluorescence in livers of animals exposed to PPD-immunization and kept on drug-free diet (see Figure 4(a)). These cells may represent a set of not fully-committed Th1 cells, thus exhibiting IL-4^+^/IFN-$\gamma^{+++}$ phenotype. Transient nature of the systemic Th1 response caused by “spillover” of immune reactions in the lungs, occuring after injection of PPD-coupled beads, may explain the failure of liver-associated T cells to complete the acquisition of Th1-type phenotype. In PPD-immunized animals receiving DEHP-diet, however, liver associated T cells appear to have fully acquired IL-4^+++^/IFN-$\gamma^{-}$ (Th2; green) phenotype (see Figure 4(b)). Taken together, our data support the conclusion that Th1/Th2 balance in DEHP-treated animals is shifted towards Th2 phenotypic response which ameliorated the effects of the Th1-type response observed in PPD-immunized animals on drug-free diet.

*Trans*activation assays showed that monoethylhexylphthalate (MEHP), the primary metabolite of DEHP [15], activates PPARα which is abundant in the liver [8, 18], as well as PPARγ [15]. However, MEHP is 5-fold more effective as a PPARα than PPARγ agonist [15], suggesting that the DEHP-evoked effect, observed in this study, is most likely mediated through the PPARα. This conclusion is supported by findings from our laboratories showing similar results by the PPARα selective agonist, clofibrate (Shnyra and Badr, unpublished results).

The precise mechanism involved in this PPARα-mediated immune deviation remains unclear. However, it has been speculated that induction of GATA-3, a transcription factor and a master regulator of Th2 differentiation, may be involved [6]. Induction of GATA-3 by PPARα agonists may depend on STAT-6, another transcription factor associated with Th2 response [6]. Interestingly, experimental evidence suggests that immune deviation regulated by PPARα may occur, at least in part, via epigenetic mechanisms not involving DNA binding [6].

### 4.2. Potential immune-related health hazards due to PPARα activation

Despite the obvious benefit of the PPAR-mediated protection of the liver against parenchymal damage caused by Th1 response, it is noteworthy that this effect may occur at the expense of other crucial functions of the immune system.

In the United States, nearly 40,000 individuals are infected each year with the hepatitis C virus [19]. Although a predominant Th1-like response is associated with a self-limited infection [3], this response is not without potential complications. Viral hepatitis is characterized by hepatocellular necrosis, an effect which is mediated by a Th1-like response involving IFN-γ [3]. In addition, the activation of liver-associated T lymphocytes has also been reported to play a role in the pathogenesis of alcoholic hepatitis [20].

In contrast to the potentially beneficial effect of immune deviation such as that occurring upon the activation of PPARα [3, 6], and this study, upsetting the Th1/Th2 balance in favor of the Th2 response may also result in serious consequences. For example, it has been shown that immune deviation is associated with a significant enhancement in the replication, and invasion across mucosal membranes, of the HIV virus [21]. Indeed, IL-4, a cytokine characteristic of Th-2 immune responses, is critical for X4 virus replication [21]. Treatments which induced IL-4 led to highly productive viral replications in cultured macrophages and T cells, as well as in vivo; an effect which was abrogated by pretreatment with IL-4 antisense [21]. Accordingly, it can be speculated that exposure to PPARα activators may exacerbate the condition of individuals infected with the HIV virus by contributing to an *immune* environment favorable for enhanced viral replication and opportunistic infections. Furthermore, immune deviation is expected to interfere with the ability of the immune system to mount a vigorous defense against other viruses, leading to chronicity and potential hepatocarcinogenicity in the case of hepatic infections.

Another potential health hazard that may result from exposure to environmentally abundant PPAR activators, such as the phthalate plasticizers [10–15, 22], stems from the fact that Th2-associated cytokines play a role in the development of asthma; a disease which has emerged as a worldwide public health problem associated with an increased risk of morbidity and mortality [23]. Last but not least, immune deviation may also hinder the ability of the immune system to respond efficiently to vaccines, a response that seems strongly biased toward Th1 activity [23].
